# Sensory, Physicochemical, and Cooking Qualities of Instant Noodles Incorporated with Red Seaweed (*Eucheuma denticulatum*)

**DOI:** 10.3390/foods11172669

**Published:** 2022-09-01

**Authors:** Wee Yin Koh, Patricia Matanjun, Xiao Xian Lim, Rovina Kobun

**Affiliations:** 1 Faculty of Food Science and Nutrition, Universiti Malaysia Sabah, Jalan UMS, Kota Kinabalu 88400, Malaysia; 2 Seaweed Research Unit, Universiti Malaysia Sabah, Jalan UMS, Kota Kinabalu 88400, Malaysia; 3 Food Technology Division, School of Industrial Technology, Universiti Sains Malaysia, Minden 11800, Malaysia

**Keywords:** functional foods, seaweeds, noodles, ready-to-eat

## Abstract

Instant noodles are consumed worldwide, but instant noodles are often unhealthy. Therefore, in the current study, instant noodles were produced with composite flour (a blend of wheat flour and potato starch at weight ratios of 9:1, 8:2, and 7:3) incorporated with red seaweed powder (*Eucheuma denticulatum*) in proportions of 0, 5, 7.5, 10, 12.5, and 15%. The noodles’ sensory, physicochemical, and cooking properties were then determined. The incorporation of 7.5–15% of seaweed powder significantly (*p* < 0.05) increased the cooking yield, reduced the cooking loss, lengthened the cooking time, and decreased the pH values and water activity. The addition of seaweed powder weakened the tensile strength and softened the noodles. Seaweed noodles were denser and greener than control noodles. Among the three seaweed noodles (F2, F5, and F12) selected through the ranking test, panelists preferred F2 and F5 (both scoring 4.63 on a 7-point hedonic scale for overall acceptability) more than F12. Overall, F5 (at a wheat flour: potato starch ratio of 9:1; 15% seaweed powder) is the best-formulated seaweed noodle in this study, owing to its highest cooking yield and lowest cooking loss even with prolonged cooking, lowest water activity, and acceptable sensory qualities.

## 1. Introduction

Instant noodles are precooked, dried, and commercially packed noodles, which can be consumed after cooking or soaked in boiling water for 3–5 min. Instant noodles are among the most popular convenience foods consumers prefer globally due to their convenience, simplicity of preparation, relatively longer shelf life, shelf stability at room temperature, range of flavors, and reasonable cost [1,2]. According to the latest report by the World Instant Noodles Association (WINA), the global demand for instant noodles was 117 billion servings in the year 2020, and nearly 1.35% of this consumption came from Malaysia, indicating that about 4.3 million packages of instant noodles were consumed by Malaysians per day [3].

The main ingredients of instant noodles are flour (mainly wheat flour), starch, water, and salt. Consequently, instant noodles are always high in carbohydrates and fat but lack essential food components or nutrients required in the daily human diet, such as fiber, vitamins, and minerals. Hence, they are often criticized as unhealthy [4]. Owing to the low nutritional values, frequent consumption of instant noodles could also lead to malnutrition, diet, nutrient-related diseases, and cardiometabolic disorders such as obesity, hypercholesterolemia, and metabolic syndrome [1]. As long as the demand for instant noodles is still growing, developing healthier instant noodles is needed. Good quality noodles are always characterized by their firm, elastic, and chewy texture [5]. Indeed, the good textural properties of noodles are governed by the gluten protein of wheat flour [6]. While Malaysia is not a wheat-producing country, the dependence on wheat flour in noodle manufacturing should be reduced to lessen the importation of wheat and the cost of production. For instance, composite flours in noodle production have been encouraged. The extensive coastline fringed by numerous coral-surrounded islands with sandy beaches, seawater with moderate temperature and equitable salinity, rich solar radiation, and daily mean daylight of 12 h has made Malaysia one of the world’s seaweed-producing countries [7]. Medium-to-large-scale seaweed cultivation has been developed in both the coastal areas of East and West Malaysia (Langkawi, Teluk Kemang in Negeri Sembilan, Tanjung-Adang and Merambong in Johor, and Semporna in Sabah) [8]. The production of noodles with a higher nutritional value and higher quality but at a lower cost is made possible by using locally accessible seaweed as a partial substitute for wheat flour. This also increases the variety of noodles, diversifies the use of seaweed, and maximizes the Malaysian seaweed food industry.

Seaweed is a highly nutritive marine food with various health benefits [9]. Seaweed is rich in polysaccharides and has been reported to reduce hepatic lipid accumulation and prevent atherosclerosis. Seaweed is also a potential prebiotic ingredient due to its rich dietary fiber content. It has been proposed to improve digestion, relieve pain, and prevent the occurrence of dysentery, diarrhea, constipation, and colon cancer. Long-term consumption of seaweed has also been proven to reduce blood pressure and the incidence of stroke [10]. Seaweeds have also been reported to have an excellent texturizing effect because of their high hydrocolloid content. Previously, seaweed powder had effectively improved the textural properties of wheat products such as bread [11] and muffins [12]. *Eucheuma denticulatum* (red seaweed) is one of the species of seaweed cultivated in Malaysia (east coast), the Philippines, Indonesia, and East Africa. *E. denticulatum* is known to contain valuable macronutrients such as carbohydrates (24.80–69.66%), dietary fiber (13.10–19.89%), protein (6.00–12.10%), lipids (0.60–4.10%), and micronutrients such as vitamins (vitamins A, B, C, E), minerals (sodium, potassium, calcium, magnesium), polyunsaturated fatty acids, and amino acids that could exert health benefits and provide balanced nutrients to consumers [13,14,15,16]. *E. denticulatum* is a rich source of bioactive components (such as saponin, flavonoids, triterpenoid, steroid, alkaloid, tannin, and glycoside) with antioxidant activities [17]. To the best of our knowledge, no research has been done on *E. denticulatum*’s antinutrient profile. The review subtly notes that compared to brown and green seaweed, red seaweed generally has a lower quantity of anti-nutritional compounds [18]. The authors discussed that the digestibility of proteins can be impacted by processing techniques (such as hydrolysis, heating, drying, etc.) and is mainly constrained by antinutrients (i.e., chymotrypsin and trypsin inhibitors). Rawiwan et al. [18] reviewed the amino acid profile and the bioavailability of red, brown, and green seaweed dietary proteins, then concluded that red seaweed typically contains more protein and fewer antinutrient compounds than brown and green seaweed. Although seaweed has numerous health benefits, seaweed-based functional foods are still in an early developmental stage. Since instant noodle products are widely consumed worldwide, they are one of the best sources to incorporate seaweed.

Previously, studies have been published on incorporating seaweed into noodle products. Green seaweed (*Caulerpa* sp.), Cyanobacteria (Spirulina), brown seaweed (*Laminaria ochroleuca*), and red seaweed (*Kappaphycus alvarezii*) have been incorporated into the production of wheat noodles [19], yellow alkaline noodles [20], and gluten-free pasta [21,22], respectively. In general, seaweed incorporation has improved noodles’ nutritional and cooking quality [23]. Nevertheless, to the best of our knowledge, no studies have been conducted addressing red seaweed (*E. denticulatum*) in the formulation of instant noodles. Therefore, this work aimed to evaluate the potential application of red seaweed (*E. denticulatum*) powder in developing instant noodles by incorporating the seaweed powder into the instant noodle’s formulation at different levels. While the noodles’ cooking quality and physical as well as sensory properties were determined, the best-formulated noodles sample was identified.

## 2. Materials and Methods

### 2.1. Materials

Fresh red seaweed (*E. denticulatum*) originated from Semporna (Sabah, Malaysia), and was obtained from the Seaweed Research Unit (UPRL), Faculty of Science and Natural Resources, Universiti Malaysia Sabah (UMS) (Sabah, Malaysia). The wheat flour (29–33% wet gluten content) was bought from Malayan Flour Mills Berhad (Penang, Malaysia), whereas potato starch, salt, and sodium bicarbonate were purchased from Bake with Me Sdn. Bhd. (Lintas, Kota Kinabalu, Sabah, Malaysia).

### 2.2. Production of Seaweed Powder

The seaweed (stored and preserved in a cold room) was thoroughly washed under running tap water to remove the salt, epiphytes, sand, and unwanted impurities attached to the surface. After 30 min of steeping, the seaweed was rewashed with distilled water, drained, cut into pieces (3 cm), and subsequently dried in a cabinet dryer (Thermoline, Wetherill Park, NSW, Australia) at 40 °C for 24 h. The dried seaweed was then grounded by using a grinder (Orimas FFC 23, Agrowindo, Malang, Indonesia) and sieved on asieve shaker (Endecotts Ltd., London, UK) to obtain seaweed powder with a particle sizes <125 µm.

### 2.3. Experimental Design

In this study, partial substitutions of the blend of wheat flour and potato starch with red seaweed (*E. denticulatum*) powder were performed to formulate instant noodles. A 5 × 3 factorial design consisting of 5 levels of seaweed powder substitution (5, 7.5, 10, 12.5, and 15%) and three levels of wheat flour/potato starch composition (ratio of 9:1, 8:2, and 7:3) were used, thus giving a total of 15 different formulations, as shown in Table 1. A formulation of instant noodles without substituting seaweed powder was used as a control sample.

### 2.4. Preparation of Instant Noodles with Seaweed Powder

The preparation of instant noodles incorporated with seaweed was divided into three stages: (1) mixing raw materials, (2) mixing, resting, and sheeting of dough, and (3) steaming and drying of noodles [24].

#### 2.4.1. Mixing of Raw Materials

The wheat flour, potato starch, salt, and sodium bicarbonate were pre-dissolved in distilled water, and seaweed powder mixed in a dough mixer (N-50, Hobart, North York, Ontario, Canada).

#### 2.4.2. Mixing, Resting, and Sheeting of Dough

The dough was kneaded into a round cushion shape and covered with a plastic film. After resting for 20 min at room temperature (25 °C), the dough was re-kneaded, rounded, and sheeted on a pasta maker (Marcato Ampia, Model 150, Campodarsego PD, Italy) with the roller gap set to 2.5 mm until number 8 settings of pasta maker to obtain dough sheet with a thickness of 0.9 mm. The dough sheet was then cut into noodle strips with a diameter of 1.5 mm.

#### 2.4.3. Steaming and Drying of Noodles

The noodles were steamed at 100 °C for 20 min using a food steamer (Pensonic, PSM-160, Mississauga, ON, Canada). After steam cooking, the noodle strips were cut (single serving size) and molded into a block. The noodles were then dried in a drying cabinet (SHIN-1 CO/9R, Kawasaki, Japan) for 4 h at 50 °C (inverted every one hour) until the product reached a moisture content and water activity of 8–12% 0.6–0.8, respectively. After being cooled for one hour at room temperature (25 °C), the dried noodles were sealed in polypropylene (PP) plastic (18 cm × 11cm, thickness: 0.1 mm) until used for analysis.

### 2.5. Physicochemical and Cooking Qualities

#### 2.5.1. Cooking Quality

##### Cooking Yield

The cooking yield of noodles was measured according to the AACC method [25]. About 10 g of instant noodles were weighed and cooked in 150 mL boiling distilled water (100 °C) for 10 min under continuous magnetic stirring in a covered beaker. Subsequently, the cooked noodles were removed from the boiling water, rinsed in cold water, drained, and left to cool for 15 min at room temperature (25 °C) before being weighed. The cooking yield of the noodles was calculated using the equation Equation (1) below:

(1) $$\mathrm{Cooking}\;\mathrm{yield}\;\left(\%\right)=\frac{W_{A}}{W_{B}}\times 100$$

where W_A_ is the weight of the cooked noodles and W_B_ is the initial weight of the noodles.

##### Cooking Loss

The cooking loss of noodles was assessed according to the AACC method [25]. The water used to cook the noodles was transferred to a 250 mL volumetric flask and diluted to the mark with distilled water. After the solution was mixed thoroughly, a 10 mL aliquot was pipetted into an aluminium dish and dried in an oven at 105 °C until a constant weight was attained. The cooking loss of the noodles was calculated using the Equation (2) below:

(2) $$\mathrm{Cooking}\;\mathrm{loss}\;\left(\%\right)=\frac{W_{R}}{W_{B}}\times 100$$

where, W_R_ is the weight of dried residue in cooking water and W_B_ is the initial weight of the noodles.

##### Optimum Cooking Time

The cooking time of noodles was determined according to the method proposed by Prerana and Anupama [2] with slight modification. The noodles were cooked in distilled water at 100 °C (10 g/1000 mL). After the noodles were boiled for 2 min, a noodles strip was withdrawn, rinsed, and pressed between two transparent glass plates every 15 s to observe the presence of the white core. The optimal cooking time was achieved when the noodles strip’s rigid and white center part became invisible.

#### 2.5.2. Textural Quality

Noodles’ texture properties (tensile strength and hardness) were examined using a texture analyzer (TA-XT2i, TMS-Pro, Food Technology Corporation, Sterling, VA, USA). All the noodles samples were cooked (cooking condition: 30 g of noodles/1 L of distilled water, 100 °C, optimum cooking time), tossed, placed in a lid-covered aluminum dish at room temperature (25 °C) and subjected to texture analysis precisely 10 min after cooking [25].

Noodles (10 cm) were tied to the spaghetti tensile grips (A/SPR) probe equipped on the texture analyzer fitted with a 5 kg load cell in the tensile strength test. The test was conducted at a constant crosshead speed (3 mm/s). The tensile strength (g) was the maximum force value required to break the noodles.

The compression test measured the hardness of noodles. The noodles (4 cm) were laid side by side on the middle of the heavy-duty platform of the texture analyzer, and a total of three parts of their surface (both ends and center part) were compressed by using a 35 mm cylindrical probe (P/35), 5 kg load cell, and a crosshead speed of 2 mm/s. The hardness (g) was measured as the maximum force attained during the noodles’ surface compression.

#### 2.5.3. pH Measurement

To determine the pH values of the noodles, approximately 2 g of dried noodles and 10 mL of distilled water were homogenized by using a stomacher (Bagmixer R400, Interscience, Saint Nom, France). The pH values of the homogenized noodles samples were then determined by using a pH meter (Eutech Instruments CyberScan pH 1500, Eutech Instruments Pte Ltd., Ayer Rajah Crescent, Singapore).

#### 2.5.4. Color Measurement

A total of 15 g of dried noodles were grounded into a powder using a mortar and pestle. The color of the powdered noodles was then measured with a colorimeter (Konica Minolta CR-100, Tokyo, Japan) using the CIE 1976, L* (lightness), a* (redness/greenness), and b* (yellowness/blueness) color scale.

#### 2.5.5. Water Activity Measurement (a_w_)

The water activity of the noodles (powdered, 1.5 g) was examined by using a water activity meter (HygroLab 3, Rotronic, Switzerland).

### 2.6. Sensory Evaluation

Ranking and hedonic tests were conducted in this study. Both tests were conducted in the individual sensory booths (25 °C) in the sensory laboratory of the Faculty of Food Science and Nutrition, Universiti Malaysia Sabah (UMS). All respondents have consented to participation in the study. According to Tan et al. [26], the seaweed instant noodles were cooked before sensory procedures. The noodles were cooked in boiling water at a noodles: water ratio of 1:10 (*v*/*w*) according to their optimum cooking time and served (a total of three strands of noodles with a length of 10 cm) to the panelists immediately after cooking, in a plastic container (30 cm^3^) coded with a random 3-digit code. Drinking water was also given to the panelists to rinse their mouths in between each evaluation.

#### 2.6.1. Ranking Test: Balanced Incomplete Block Design (BIBD)

A balanced incomplete block design (BIBD) was used to design the sensory evaluation (t = 15, b = 35, k = 3, r = 7, λ = 1). A total of 105 panelists (students from the Faculty of Food Science and Nutrition, UMS) who have basic knowledge of sensory assessment were involved in this ranking sensory assessment. Each panelist was given three samples at each session and was asked to rank their degree of overall acceptability of the three samples from 1 to 3, where 1 was the most preferred sample and 3 for the least preferred sample. The non-parametric Friedman test was applied to analyze the results obtained through BIBD with t-1 degrees of freedom at a 5% significant level. After that, the Fisher’s least significant difference (LSD) test was performed to analyze the pattern of difference between means. A total of 3 instant noodles with lower ranks in the BIBD test were selected (from 15 formulations) and proceeded to the hedonic test. Where t is the number of samples (instant noodles), b is the number of panelists in a block, k is the number of samples evaluated in each block (by each panelist), r is the repetitions (number of times each sample appears across all blocks, and λ is the number of times each pair of instant noodles appeared in the same block.

#### 2.6.2. Hedonic Test

The hedonic test on instant noodles was conducted according to the methods described by Tan et al. [26] and Llavata et al. [27]. Trained panelists, as many as 40 students from the Faculty of Food Science and Nutrition, UMS were recruited to evaluate the color, texture (elasticity), aroma, seaweed flavor, taste, mouthfeel, saltiness, stickiness, and overall acceptability of the noodles samples using a 7-point hedonic scale. At this moment, the score of 1 represents dislike extremely, 4, neither like nor dislike, and 7, like extremely.

### 2.7. Statistical Analysis

Statistical analyses of all the experimental data (except for the ranking test involved in sensory evaluation) obtained were performed by using SPSS (Statistical Package for the Social Sciences Version 17.0, SPSS Inc., Chicago, IL, USA), using the one-way analysis of variance (one-way ANOVA) and post hoc Tukey’s Honestly Significant Difference (HSD) test at a significance level *p* < 0.05. All the data were reported using mean values of the triplicate determinations ± standard deviation. The significant differences among the ranking data (sensory evaluation) were evaluated by the non-parametric Friedman test followed by Fisher’s Least Significant Difference (LSD) test, both *p* < 0.05.

## 3. Results and Discussion

### 3.1. Physicochemical and Cooking Qualities

#### 3.1.1. Cooking Quality

##### Cooking Yield

**Figure 1:**
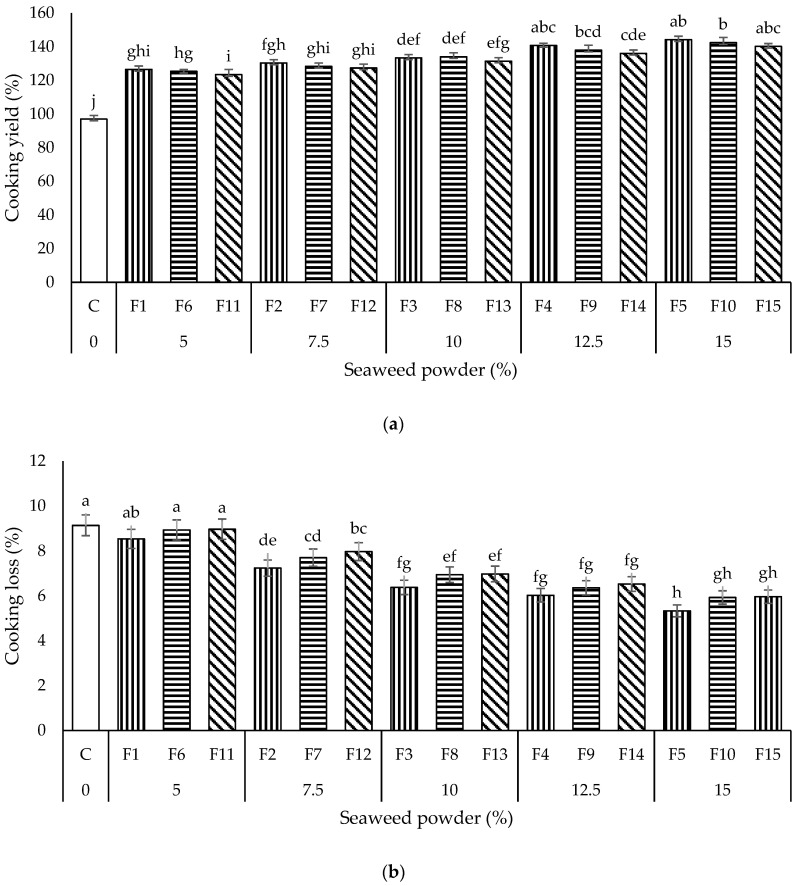

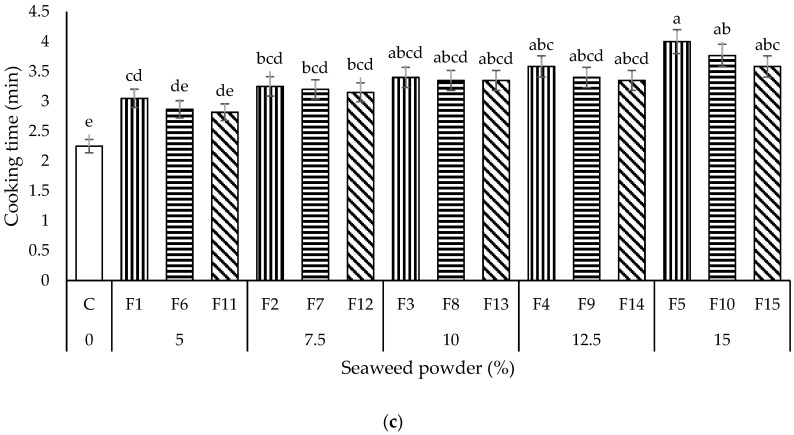
Cooking quality (**a**) cooking yield, (**b**) cooking loss, and (**c**) cooking time of all the noodles samples. Data are presented as mean ± standard deviation (*n* = 3). ^a–e^ with different superscripts indicate the significant difference among the noodle samples (Tukey’s HSD, *p* < 0.05). Bars with vertical, horizontal, and diagonal patterns = wheat flour:potato starch ratios of 9:1, 8:2, and 7:3, respectively.

The cooking yield is the ability of dried noodles to absorb water from the cooking medium when cooked. The cooking yield of all the noodles samples is shown in Figure 1a. From the results obtained, the cooking yield of all the seaweed noodles ranged from 123.44 to 144.18% and was significantly higher (*p* < 0.05) than the control sample (C, 97.00%). The cooking yield of noodles increased with the incorporation of SP. Seaweeds have been reported to absorb and retain water effectively due to their rich hydrocolloid and polysaccharide content [23]. Our findings were consistent with a recent study by Agusman and Wahyuni [19]. In this study, the sodium bicarbonate content of each sample of noodles was 0.3%. The presence of sodium bicarbonate could increase the competition for water between gluten protein and starch in wheat flour in the noodle system and delay wheat flour hydration. As the control noodle sample (C) did not contain SP with a high water absorption capacity, it was unable to absorb a significant amount of water during cooking, resulting in a low cooking yield.

Among all the seaweed noodles, the sample with the highest cooking yield was F5 (144.18%), containing 15% seaweed powder (SP). Compared to the control (C), incorporating 15%, SP remarkably increased the cooking yield by 47.18%. Furthermore, the cooking yield of seaweed noodles was also observed to increase (*p* > 0.05) with the amount of SP incorporated (F1 < F2 < F3 < F4 < F5; F6 < F7 < F8 < F9 < F10; F11 < F12 < F13 < F14 < F15). These implied that the cooking yield of noodles could be increased with the amount of SP incorporated as more water could be absorbed.

Although no significant differences were observed, the cooking yield of the noodles samples formulated using different wheat flour (WF) and potato starch (PS) blending ratios (9:1, 8:2, 7:3) were observed to decrease gradually with the increment of PS. The results indicated that the addition of PS to the formulation decreased the cooking yield of noodles. The decrement in the cooking yield of noodles could be due to the loss of starch. Futhermore, the increase in PS content may reduce the cohesiveness of noodles and subsequently weaken and loosen the noodles’ inner structure, resulting in a lower cooking yield [28].

##### Cooking Loss

Cooking loss is the measure of the number of solid components leaching into the cooking water; which indicates the extent of noodles’ damage as well as the ability of noodles to maintain their structural integrity while cooking in hot water; therefore, cooking loss is commonly used to describe the overall quality of noodles [20]. Noodles with good cooking quality must not have a cooking loss of more than 10% [22]. Since the cooking loss of all the noodles in this study was between 5.33 and 9.14%, the noodles samples possessed good cooking quality

Figure 1b demonstrates the cooking loss of all the noodles samples. The cooking loss was observed to decrease significantly (*p* < 0.05) with the increase of SP from 7.5 to 15% in the formulation compared to the control sample (C). Nevertheless, results revealed that incorporating 5% of SP did not significantly decrease (*p* > 0.05) the cooking loss of noodles. The results revealed that incorporating 7.5, 10, 12.5, and 15% SP in noodles reduced the cooking loss. This could be due to the seaweed carrageenan’s high water absorption ability, which enables more water uptake into the noodles’ gelatinized matrix structures [29]. The SP amount of 5% might be too small to decrease the cooking loss of noodles significantly.

Although the results were insignificant, the cooking loss of the noodles increased with the amount of PS used (WF: PS ratio of 9:1 < 8:2 < 7:3). The addition of PS negatively affected the cooking quality of noodles. As the amount of wheat gluten protein decreased, the overall structure of the protein-starch matrix was weakened, resulting in noodles with a fragile structure. Consequently, more soluble components of the noodles leached into the cooking water. The findings concur with Tao et al. [28], who also discovered that replacing WF with PS increased cooking loss. Noodles with good cooking quality usually have a high cooking yield and a low cooking loss. In this study, noodles sample F5 recorded the highest cooking yield and lowest cooking loss among all the noodles. Results suggested that the noodles in sample F5 had the highest cooking quality among all the noodles.

##### Optimum Cooking Time

The optimum cooking time for noodles is when the noodles are completely gelatinized. The optimum cooking time of all the noodles samples is presented in Figure 1c. SP incorporated noodles’ cooking times ranged from 2.82 to 4.00 min, with noodle sample F5 having the highest cooking time. Regarding sustainability, noodles with a short cooking time are preferred [29]. Despite this, according to the Malaysia Standard MS 526-2009 [30], the maximum cooking time for instant noodles is 4 min, and hence, the cooking times of all our seaweed noodles samples are acceptable.

All the seaweed noodles, except for noodles F6 and F11, required significantly (*p* < 0.05) more extended cooking time. The incorporation of seaweed powder increased the optimum cooking time of noodles. The optimum cooking times of seaweed noodles were also observed to increase (*p* > 0.05) with the amount of SP incorporated into the noodles’ formulation (F1 < F2 < F3 < F4 < F5, F6 < F7 < F8 < F9 < F10, and F11 < F12 < F13 < F14 < F15). The cooking time of the noodles was governed by the rate of water ingress in the noodles. Seaweed is rich in hydrocolloid polysaccharides, agar, alginates, and carrageenan, with high water absorption capacities. Hence, when a higher amount of seaweed is added, more water will be absorbed, resulting in higher water uptake and increased cooking time [22].

When comparing the noodles with the same SP content, the seaweed noodles with a WF:PS ratio of 9:1 required a longer time to cook than those with a WF:PS ratio of 8:2 and 7:3. The addition of PS reduced the optimum cooking time of noodles. PS had a lower gelatinization temperature than WF. Therefore, when noodle samples with a higher amount of PS were cooked in boiling water, PS would swell faster; hence, the cooking time was shortened. Our results were in good correlation with those of Parvathy et al. [24], who reported that noodles with a higher PS level are positively related to a shorter optimum cooking time.

#### 3.1.2. Textural Quality

The instrumental textural properties effectively predict and elucidate the texture-related sensory acceptability of consumers toward the noodles. Noodles with good quality shall possess a value of tensile strength and hardness [5]. Therefore, a texture analyzer was utilized to measure the tensile strength and hardness of the cooked control and seaweed instant noodles.

**Figure 2:**
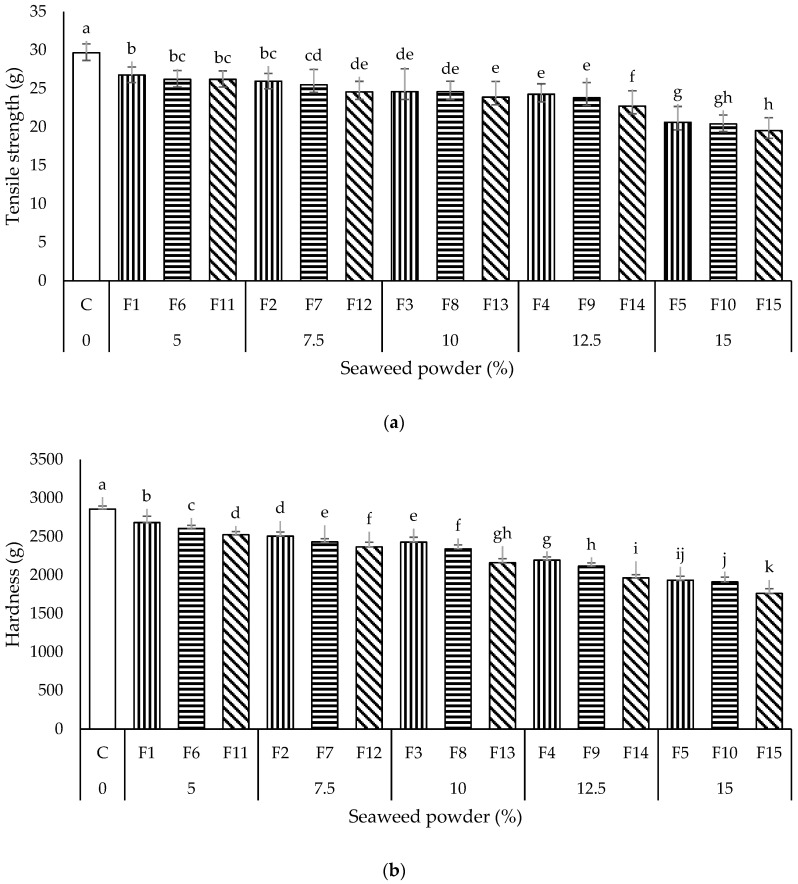
Texture properties (**a**) tensile strength and (**b**) hardness of all the noodles samples. Data are presented as mean ± standard deviation (*n* = 3). ^a–k^ with different superscripts indicate the significant difference among the noodles samples (Tukey’s HSD, *p* < 0.05). Bars with vertical, horizontal, and diagonal patterns = wheat flour: potato starch ratios of 9:1, 8:2, and 7:3, respectively.

Tensile strength is indicated by the maximum force required to snap the noodle’s strand, representing the elasticity and crushing strength of noodles [6]. The tensile strength of all the seaweed noodles (19.53–26.78) was significantly lower (*p* < 0.05) than the control noodle sample (C; 29.65 g) (Figure 2a). Although the tensile strength between noodles samples F2 and F3, F3 and F4, F6 and F7, F7 and F8, F8 and F9, F12 and F13 were statistically similar, when the ratio of WF to PS was constant, the tensile strength of the noodles decreased with the amount of SP. The decrement in the tensile strength was thought to be due to the weakening of starch-protein interaction, presumably caused by the interference between the SP and gluten protein in the noodles system. The high-water absorption capacity could cause the SP to swell significantly, resulting in structure disintegration and weakening the noodles’ tensile strength and elasticity [6].

Hardness is the force required to penetrate through the noodles with teeth during the first bite. All the seaweed noodles (1762.45–2683.10 g) showed remarkable lower (*p* < 0.05) hardness values than C (2856.45 g) (Figure 2b). On the increasing amount of SP, the hardness of seaweed noodles also showed a decreasing pattern (*p* < 0.05). The incorporation of SP led to a significant reduction in the hardness of noodles, which was thought to be due to the hygroscopic properties of seaweed. Seaweed could absorb a large quantity of water during cooking owing to its high-water absorption capacity, resulting in noodles with a soft and spongy texture [20]. Debbarma et al. [31] also reported a decrease in the hardness of noodles made using seaweed.

The hardness of all the seaweed noodles was also observed to decrease when the amount of PS used to replace WF in the formulation was increased (hardness of seaweed noodles at a WF:PS ratio of 9:1 > 8:2 > 7:3). This could be owing to the comparable lower amylose content of PS. When a high concentration of PS is included in the WF/PS blend, a lower total amylose content will be obtained, resulting in the formation of a softer gel and lower hardness of the noodles samples in this study [5]. The high addition level of the PS also increased the competition of water between gluten and starch, subsequently reducing the availability of free water for gluten to form the gluten network structure, decreasing the overall gluten content in the noodle system and resulting in a weakened dough structure and softer noodles [28].

A strong positive correlation (r^2^ = 0.838; *p* < 0.05) was observed between instant noodles’ tensile strength and hardness. This indicated that a higher tensile strength confers a more complex texture in seaweed noodles. The increasing amount of *E. denticulatum* SP could cause the instant noodles to become softer and have weaker tensile strength. Nevertheless, the comparable lower tensile strength and hardness of seaweed noodles than the control noodles indicated that *E. denticulatum* SP could not be used as a material to strengthen the structural network of the instant noodles.

#### 3.1.3. pH Measurement

**Figure 3:**
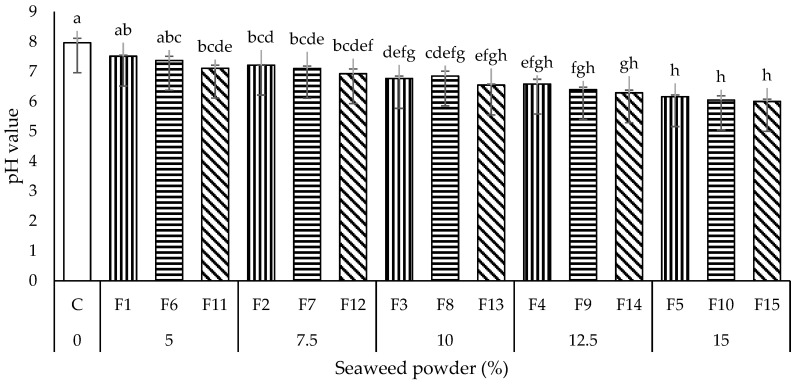
pH measurement of all the noodles samples. Data are presented as mean ± standard deviation (*n* = 3). ^a–h^ with different superscripts indicate the significant difference among the noodles samples (Tukey’s HSD, *p* < 0.05). Bars with vertical, horizontal, and diagonal patterns = wheat flour: potato starch ratios of 9:1, 8:2, and 7:3, respectively.

The pH values of all the noodle samples are presented in Figure 3. The pH values of all the seaweed instant noodles samples were 6.0 to 7.52, lower than the control ones (C; 7.96). All the seaweed noodles were statistically different (*p* < 0.05) from C except for F1 and F6. The incorporation of SP increased the noodles’ acidity. This might be due to the seaweed’s glutamic and aspartic acid residues [32]. These results align with the research conducted by Shahsavani and Mostaghim [20], where lower pH values were also reported in the yellow alkaline noodles after the addition of seaweed.

#### 3.1.4. Color Measurement

**Figure 4:**
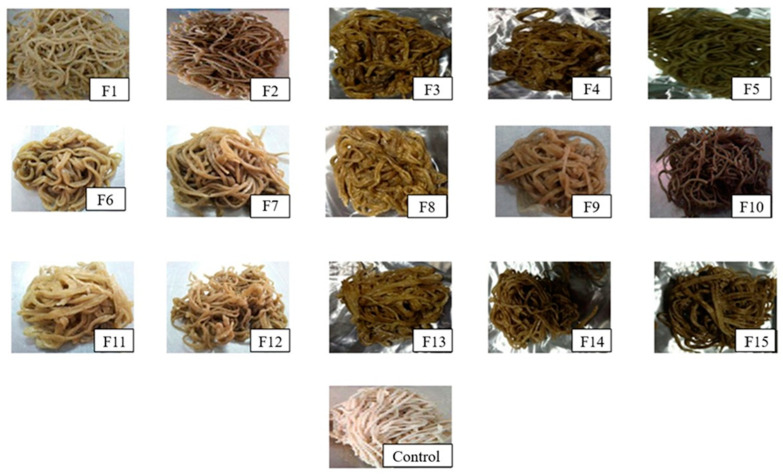
Appearances of all the instant noodles incorporated with seaweed powder and the control noodles (without seaweed powder).

**Figure 5:**
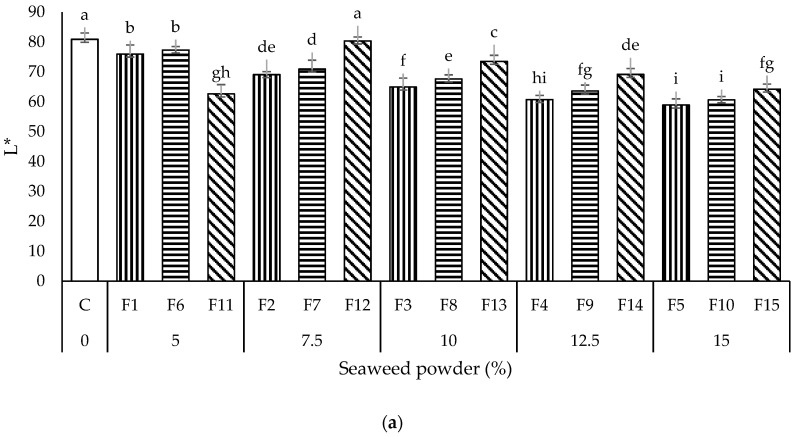

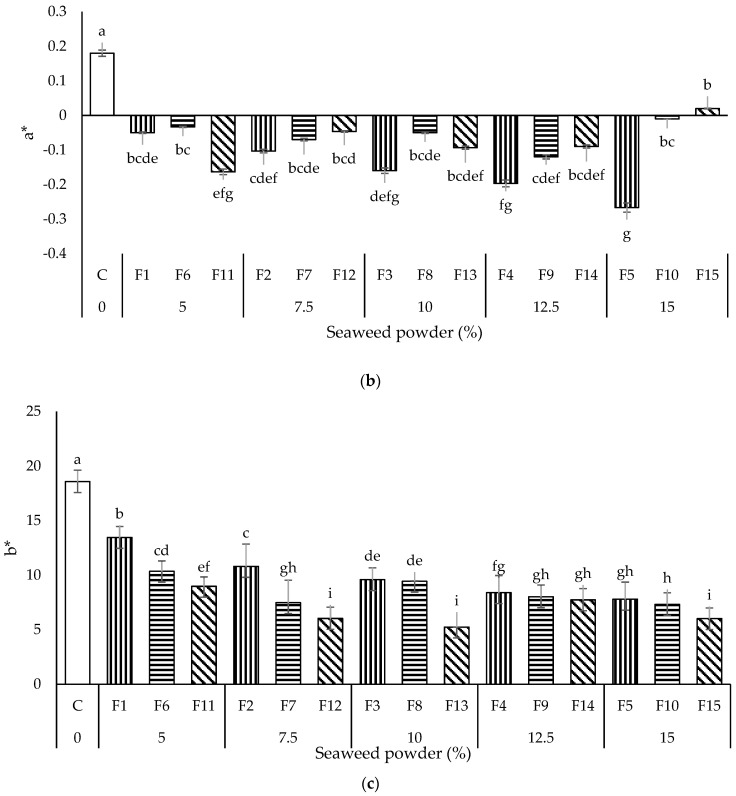
Color measurement (**a**) L* (lightness), (**b**) a* (redness/greenness), and (**c**) b* (yellowness/blueness) of all the noodles samples. Data are presented as mean ± standard deviation (*n* = 3). ^a–i^ with different superscripts indicate the significant difference among the noodles samples (Tukey’s HSD, *p* < 0.05). Bars with vertical, horizontal, and diagonal patterns = wheat flour: potato starch ratios of 9:1, 8:2, and 7:3, respectively.

Color and appearance are important quality criteria for consumer acceptance in noodles products [21]. The appearances and color profiles of all the noodles samples prepared in this study are shown in Figure 4 and Figure 5, respectively. The control noodles (without seaweed) were visually observed to be the whitest, whereas the seaweed noodles varied in color from pale green to dark green (Figure 4).

In general, all the formulated seaweed noodles had lower (*p* < 0.05) lightness (L* = 60.63–80.33), redness (a* = −2.67–0.02), and yellowness values (b* = 5.24–13.46), compared to the control noodles (L* = 80.88; a* = 0.18; b* = 18.53). All the L* values of seaweed noodles samples were significantly different (*p* < 0.05) from the control noodles except for noodles sample F12 (Figure 5a). Except for the noodles sample F11, the L* values of noodles samples had been observed to increase with the addition of PS. The incorporation of SP had obstructed the internal network structure of the noodles, reduced the light penetration, and hence, resulted in darker appearances [20]. Meanwhile, the increment in PS diluted the gluten protein content and weakened the noodle’s structure’s strength, thus producing noodles with a lighter color [33]. The addition of SP had remarkably (*p* < 0.05) reduced a* values compared to the control noodles, C. Among all the noodles samples, only noodles samples C and F15 showed a positive a* value (Figure 5b). The results showed that noodles sample F15 demonstrated more redness and less greenness than other seaweed noodles. The colors of noodles were closely related to the pigment composition in the seaweed and the processing procedures of the noodles. The redness color of F15 was provided by the phycocyanin, phycoerythrin, chlorophyll a, and xanthophyll pigments in red seaweed [23]. The low redness values of other seaweed noodles were probably due to the pigment’s degradation during the drying process [21]. The yellowness (b*) of noodles had also been observed to reduce (*p* < 0.05) with the incorporation of SP and decrease (*p* > 0.05) with the amount of PS in the formulations, from WF:PS ratio of 9:1 > 8:2 > 7:3 (Figure 5c). The decline in the yellowness of seaweed noodles was due to the discoloration of SP caused by the drying process during the noodles production and the higher clarity of PS compared to WF, respectively [34]. Decrement in L*, a*, and b* values of noodles after the incorporation of seaweed was also reported by Shahsavani and Mostaghim [20], Debbarma et al. [31], and Sholichah et al. [22].

#### 3.1.5. Water Activity Measurement (a_w_)

**Figure 6:**
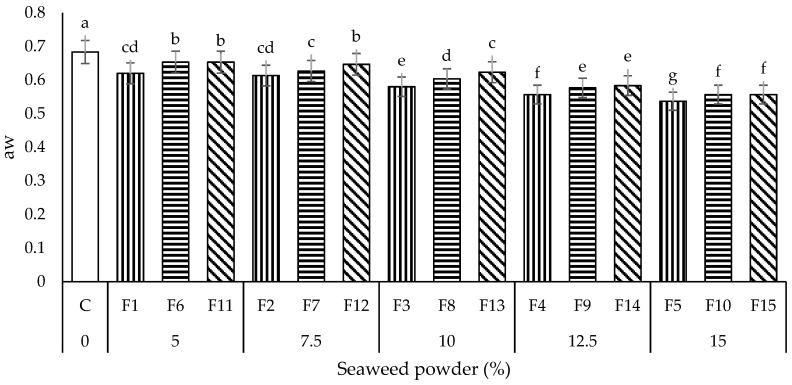
Water activity measurement (a_w_) of all the noodles samples. Data are presented as mean ± standard deviation (*n* = 3). ^a–g^ with different superscripts indicate the significant difference among the noodles samples (Tukey’s HSD, *p* < 0.05). Bars with vertical, horizontal, and diagonal patterns = wheat flour: potato starch ratios of 9:1, 8:2, and 7:3, respectively.

Water activity (a_w_) is the measurement of unbound water in a product and an indicator of food safety and quality. The a_w_ value below 0.6 could prevent the spoilage of dried food by inhibiting microbial growth and slowing down most enzymatic and chemical reactions [19]. All the seaweed instant noodles samples exhibited significantly lower (*p* < 0.05) water activities (a_w_; 0.54–0.65) than the control noodles sample (0.63) (Figure 6). The addition of *E. denticulatum* SP significantly lowered the a_w_ of noodles due to the SP’s high water holding capacity. The a_w_ values of noodles were also observed to decrease (*p* < 0.05) when the amount of SP in the noodles’ formulation was increased from 5% to 10%, 12.5%, and 15%. As the amount of SP incorporated increases, more flour particles are available to attract and hold water in the noodles system, thus resulting in lower a_w_ [15].

When the noodles with a similar amount of SP (10%) were compared, the a_w_ values of noodles samples were increased in the order: F3 < F8 < F13. It is well evident from Figure 6 that the water activity significantly increased (*p* < 0.05) with an increase in PS content in the noodles’ formulation. PS exhibited weaker water-binding properties than the gluten protein in WF. Therefore, more free unbound water remained when the amount of PS was higher than WF in the noodles system [28]. Among all the noodle samples in this study, noodle sample F5 (at a WF: PS ratio of 7:3, SP content of 15%) had the lowest (*p* < 0.05) a_w_ (0.54), which fell below 0.60. With the lowest a_w_, F5 was expected to be the most durable and extended shelf life if packed and stored correctly.

### 3.2. Sensory Evaluation

#### 3.2.1. Ranking Test

A ranking test was conducted at the first stage of sensory evaluation to select the best three formulations out of 15 formulations using BIBD. According to the results obtained from balanced incomplete block design (BIBD), the most preferred noodles sample was F2 (mean rank score: 3.71), followed by F12 (4.38), F5 (4.50), F1 (5.86), F4 (6.50), F3 (7.21), F7 (8.05), F9 (8.07), F8 (8.67), F10 (9.38), F13 (10.19), F14 (10.81), F6 (10.83), F15 (10.88), and the least preferred, F11 (10.95). Overall, according to the data obtained (F2 < F5 (at a WF: PS ratio of 9:1); F7 < F10 (8:2); F12 < F15 (7:3); SP content of F2, F7, F12 = 7.5%, F5, F10, F15 = 15%), the panelists preferred noodles containing a moderate percentage of 7.5% SP, followed by the highest percentage, 15% of SP. When eaten, F2 with lower PS content had better texture properties in chewiness and hardness. A higher PS content tended to produce softer noodles. Consumers have varying preferences when it comes to texture. In our case, the more delicate noodles such as F11 were not desirable for our panelists. The high sensory acceptance of F2 was also associated with its acceptable pale greenish color (Figure 4) due to the incorporation of SP in moderate content. Also, F2 did not possess rough surfaces and gritty mouthfeel. The panelists preferred noodles with 7.5% SP the most, followed by the noodles with 15% SP. This was probably due to the less sticky texture of noodles with 15% (double amount) SP compared to 7.5%. The addition of SP could reduce the stickiness of noodles. The noodles formulated with 7.5 and 15% SP generally achieved satisfactory overall acceptances in all sensory attributes.

**Figure 7:**
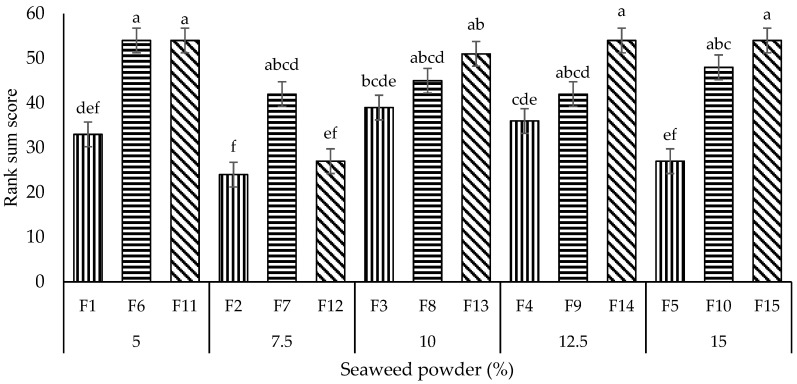
Rank sum scores for all the noodles samples. Lower rank-sum scores indicate a higher overall preference, whereas higher rank-sum scores indicate a lower preference. ^a-f^ with different superscripts show the significant difference among the noodles samples (Friedman test, LSD, *p* < 0.05). Bars with vertical, horizontal, and diagonal patterns = wheat flour: potato starch ratios of 9:1, 8:2, and 7:3, respectively.

Based on the Friedman and LSD test in Figure 7, the noodles sample F2 received the lowest mean rank score among all 15 formulations. It was remarkably (*p* < 0.05) more preferred by the panelists compared to the noodles samples F3, F6, F7, F8, F9, F10, F11, F13, F14, and F15. The noodles sample with the highest mean rank score (least preferred by the panelists), F11, on the other hand, showed no significant differences between noodles samples F6, F7, F8, F9, F10, F11, F13, F14, and F15. Hence, the noodles samples F3, F6, F7, F8, F9, F10, F11, F13, F14, and F15 were not considered for the subsequent hedonic test. The noodles with the second, third, fourth, and fifth lowest mean rank scores after F2 were F12, F5, F1, and F4, respectively. Noodles samples F1 and F4 with higher mean rank scores than F2, F5, and F12 were not selected for the hedonic test due to their low SP content and color attribute, respectively. Noodles sample F1 (SP content: 5%) had the lowest SP content among noodles samples F1 (5%), F2 (7.5%), F4 (12.5%), F5 (15%), and F12 (7.5%).

According to the panelists, the SP with a content of 5% did not impart distinctive taste, aroma, color, and texture attributed to the noodles. On the other hand, noodles sample F4 did not possess a dark green color, giving the appearance of nutraceutical plant-derived foods made from natural vegetables as F5 did. F4 also did not show an attractive clear green color like the noodles sample F2. Therefore, noodles samples F2, F5, and F12 were selected among the 15 formulations to undergo the hedonic test.

#### 3.2.2. Hedonic Test

A total of three noodles samples (F2, F5, and F12) selected from the ranking test were involved in the hedonic test. The attributes of the noodles, such as color, texture (elasticity), aroma, seaweed flavor, taste, mouthfeel, saltiness, stickiness, and overall acceptability, were assessed, and the results of the hedonic test are presented in Table 2.

Color plays a prominent role in capturing consumer attention, influencing consumer assessment of a product’s perceived quality and overall impression, and satisfying the human appetite. The highest mean score for the color attribute was observed in the noodles sample F2 (5.25), followed by F5 (4.55) and F12 (3.68), and they were statistically significant (*p* < 0.05) and different from each other. The color scores were observed to be affected by the formulations of the noodles, in particular, the amount of SP and the composition of WF and PS. By comparing the noodles samples F2 and F5 (both at a WF:PS ratio of 9:1), the panelists preferred noodles sample F2 with the incorporation of 7.5% SP more owing to its attractive pale green color (Figure 4). Panelists prefer seaweed noodles with unique color, which is still less available in the market. F5 formulated with a higher SP (15%) had a darker green color. The reason panelists preferred noodles samples F2 and F5 more than F12 could also be their translucent appearance. In noodles production, noodles strands with a more apparent appearance can be produced by replacing a portion of WF with PS. After cooking, noodles with transparent and shiny appearances are higher in quality and are more desirable among consumers.

Although the scores obtained were not statistically different (*p* > 0.05), noodle sample F2 scored the best (4.95) among all three noodles in terms of texture, followed by F5 (4.68) and F12, which scored the lowest (3.65). The texture of the F2 was more favored by the panelists than F5. These suggested that F5, with a higher SP (15%), possessed a lower elasticity than F2 (7.5%). Incorporating SP into the noodles system will interfere with gluten formation and weaken the elastic texture of the noodles. Therefore, the higher the SP added to noodles, the less flexible it is. According to the textural properties results, noodles sample F12 had the softest texture and lowest elasticity (*p* < 0.05), owing to its higher PS content than F2 and F5. The texture properties of noodles, especially elasticity, are strongly associated with the gluten matrix network formed. The gluten network is weakened by replacing WF with PS with lower protein content and loses elasticity. The replacement of a higher percentage of PS with lower amylose content in wheat noodles was also one of the reasons for producing noodles with a softer texture.

Seaweeds have a solid and characteristic aroma. Therefore, the addition of SP into noodles provided added flavor. From the results obtained for the aroma attribute, the noodles sample with the highest mean score was F2 (4.43), followed by F5 (4.18) and F12 (3.73). However, the score obtained by F5 was not statistically different from F2 and F12, indicating that the aroma of the noodles was not very pronounced and was difficult to distinguish by the panelists. The smell of the seaweed in the formulated noodles was not strong. Therefore, only specific panelists familiar with seaweed-based food could detect the slight aroma of the seaweed in the noodles.

Seaweed flavor attributes were evaluated to study consumer perceptions and the suitability of adding SP in instant noodles. F5 (3.98) was adjudged as having the wealthiest seaweed flavor, followed by F2 (3.88) and F12 (2.83). Noodles samples F2 and F5 were not statistically different (*p* > 0.05) from each other, but they had significant differences with F12 (*p* < 0.05). Panelists preferred noodles sample F5 with the highest SP (15%) because it had a more pronounced seaweed flavor among all three noodles samples. The incorporation of 7.5% SP was insufficient to provide rich flavor to the noodles.

Noodles samples F2 (3.98) and F5 (3.75) had significantly higher (*p* < 0.05) mean scores in terms of taste than F12 (2.30). The mean score indicated that the panelists neither liked nor disliked the taste of noodles samples F2 and F5 but moderately disliked the taste of F12. In general, the taste of noodles did not vary with an increasing amount of SP but was negatively affected by the increased PS content. The panelists perceived the taste of F12 as strange because it did not provide a good or similar taste given to the instant noodles available in the local market. This could be due to the soft texture of noodles sample F12. Noodles sample F12 did not meet the local tastes, in which the instant noodles in the market are often elastic and chewy.

The preference for the mouthfeel of noodles was rated in order of F2 (4.25) > F5 (3.58) > F12 (2.70), and they were statistically different from each other (*p* < 0.05). The results revealed that panelists neither liked nor disliked the mouthfeel of noodles F2 and F5 but disliked the mouthfeel provided by F12. These were most probably due to the texture properties of noodles. Compared to noodles sample F2, F5 with a higher seaweed powder (15%) gave a rough and grainy mouthfeel, whereas F12 with a higher PS provided a softer texture. The panelists perceived these as less desirable; hence, F2 scored the highest among the three formulations.

The saltiness attribute was considered in this sensory assessment to study the potential application of SP as a natural salt in the production of seaweed instant noodles. All three noodles samples did not vary significantly (*p* > 0.05) in their scores for the saltiness attribute. Although the noodles samples contained different SP amounts (F2, 7.5%; F5, F12, 15%), panelists thought their saltiness was similar. This could have been because the seaweed noodles samples contained less salt. The salt contained in fresh seaweed had been isolated when the seaweed was dried. The drying process of seaweed noodles and the cooking process further reduced the salt compounds in noodles, resulting in low saltiness. In conclusion, in the production of seaweed instant noodles products, seaweed cannot be used alone to provide the taste of saltiness. Some panelists suggested that salt be added to enhance the salty taste in the noodles samples.

The stickiness of noodles can be observed through their appearance, where the noodles with low stickiness give a uniform appearance and are not clumpy. Noodles with sticky and clumpy appearance are considered poor in quality. Noodles sample F2 scored comparatively higher (*p* < 0.05) in stickiness (4.83) than F5 (4.05) and F12 (3.75). The mean score results showed that the panelists liked the noodles sample F2 (at a WF:PS ratio of 9:1; 7.5% SP), neither liked nor disliked F5 (9:1; 15%) but opposed F12 (7:3; 7.5%), in terms of their stickiness. The partial replacement of WF with SP could reduce the stickiness of noodles [12]. However, a higher PS content could increase the stickiness of noodles.

For the overall acceptability, both noodles samples F2 and F5, had significantly higher (*p* < 0.05) mean scores (both scored 4.63) than F12 (3.60). Overall, the panelists preferred noodles samples F2 and F5 more than F12. The remarkably higher overall acceptability scores of noodles samples F2 and F5 can be explained through their stronger (*p* < 0.05) mean scores obtained in the attributes of color, texture (elasticity), seaweed flavor, taste, and mouthfeel as compared to F12.

## 4. Conclusions

The incorporation of red seaweed (*E. denticulatum*) powder (SP) enhanced the cooking qualities of noodles by increasing the cooking yield and decreasing the cooking loss. The incorporation of SP also reduced the pH value and water activity of noodles and darkened the color of noodles, where noodles with SP were greener. Nevertheless, the addition of SP negatively affected the texture properties of noodles. Among all the noodles samples, F5 (at a WF: PS ratio of 9: 1; 15% SP), with the highest cooking yield and lowest cooking loss, was the best noodles sample in terms of cooking quality. Among the three noodles samples (F2, F5, and F12) that had been selected through the BIBD ranking test, noodles samples F2 and F5 were preferred by the panelists over F12 (*p* < 0.05). F2 scored highest (*p* < 0.05) for the sensory attributes: color, stickiness, and mouthfeel, but it did not differ significantly from F5 in terms of aroma, texture, seaweed flavor, and taste (*p* > 0.05). After considering all the properties, seaweed noodles sample F5 was determined to be the best formulation in this study. SP as a food ingredient in noodles development offers a lot of potential; however, texture enhancement must be considered.

## Figures and Tables

**Table 1:** Formulation of noodles with the variation of seaweed powder content.

Formulations	Ingredients (%)				
	WF: PS (Ratio)	SP	Water	Sodium Bicarbonate	Salt
C (Control)	64.7 (9:1)	0	34	0.3	1
F1	59.7 (9:1)	5	34	0.3	1
F2	57.2 (9:1)	7.5	34	0.3	1
F3	54.7 (9:1)	10	34	0.3	1
F4	52.2 (9:1)	12.5	34	0.3	1
F5	49.7 (9:1)	15	34	0.3	1
F6	59.7 (8:2)	5	34	0.3	1
F7	57.2 (8:2)	7.5	34	0.3	1
F8	54.7 (8:2)	10	34	0.3	1
F9	52.2 (8:2)	12.5	34	0.3	1
F10	49.7 (8:2)	15	34	0.3	1
F11	59.7 (7:3)	5	34	0.3	1
F12	57.2 (7:3)	7.5	34	0.3	1
F13	54.7 (7:3)	10	34	0.3	1
F14	52.2 (7:3)	12.5	34	0.3	1
F15	49.7 (7:3)	15	34	0.3	1

SP = seaweed powder; WF = wheat flour; PS = potato starch.

**Table 2:** Sensory data for noodles samples F2, F5, and F12.

Noodles	Color	Texture (Elasticity)	Aroma	Seaweed Flavor	Taste	Mouthfeel	Saltiness	Stickiness	Overall Acceptability
F2	5.25 ± 1.24 ^a^	4.95 ± 1.32 ^a^	4.43 ± 0.93 ^a^	3.88 ± 1.38 ^a^	3.98 ± 1.54 ^a^	4.25 ± 1.43 ^a^	3.78 ± 1.37 ^a^	4.83 ± 1.08 ^a^	4.63 ± 1.23 ^a^
F5	4.55 ± 1.60 ^b^	4.68 ± 1.46 ^a^	4.18 ± 1.13 ^ab^	3.98 ± 1.17 ^a^	3.75 ± 1.28 ^a^	3.58 ± 1.43 ^b^	3.95 ± 1.36 ^a^	4.05 ± 1.38 ^b^	4.63 ± 1.19 ^a^
F12	3.68 ± 1.39 ^c^	3.65 ± 1.33 ^b^	3.73 ± 1.15 ^b^	2.83 ± 1.01 ^b^	2.30 ± 1.18 ^b^	2.70 ± 1.04 ^c^	3.35 ± 1.21 ^a^	3.75 ± 1.10 ^b^	3.60 ± 1.26 ^b^

Mean (±SD) values with a different superscript (^abc^) in the same column were significantly different (Tukey’s HSD, *p* < 0.05).

## Data Availability

Data is contained within the article.
